# Diversity, Host Plants and Potential Distribution of Edible Saturniid Caterpillars in Kenya

**DOI:** 10.3390/insects12070600

**Published:** 2021-07-01

**Authors:** Elizabeth Siago Kusia, Christian Borgemeister, Fathiya M. Khamis, Robert S. Copeland, Chrysantus M. Tanga, Fidelis Levi Ombura, Sevgan Subramanian

**Affiliations:** 1 International Centre of Insect Physiology and Ecology (*icipe*), Nairobi 00100, Kenya; ekusia@icipe.org (E.S.K.); fkhamis@icipe.org (F.M.K.); rcopeland@icipe.org (R.S.C.); ctanga@icipe.org (C.M.T.); lombura@icipe.org (F.L.O.); 2 Centre for Development Research (ZEF), University of Bonn, 53113 Bonn, Germany; cborgeme@uni-bonn.de; 3 Department of Entomology, National Museum of Natural History, Smithsonian Institution, Washington, DC 20013, USA

**Keywords:** *Bunaea alcinoe*, *Cirina forda*, *Gonimbrasia zambesina*, *Gonimbrasia belina*, saturniids, edible insects, entomophagy, edible caterpillars, host plants

## Abstract

**Simple Summary:**

Edible insects are a traditional food source with economic benefits in sub-Saharan Africa. Caterpillars are the most popular edible insects in this region. We focus on caterpillars in the family Saturniidae. Saturniids are big colorful caterpillars with spines on their bodies, usually found in shrubs and trees. They are rich in proteins, vitamins, and minerals. Despite their economic importance, little is known about their diversity, host plants, distribution, and potential effect of climate change on edible saturniid caterpillars in Africa. The aim of this study is to identify edible saturniids, their host plants, their current distribution and to predict the possible effects of climate change on their distribution. We documented seven species of edible saturniids namely *Gonimbrasia zambesina*, *Gonimbrasia krucki*, *Bunaea alcinoe*, *Gonimbrasia cocaulti*, *Gonimbrasia belina*, *Gynanisa nigra* and *Cirina forda*. These caterpillars mostly occur twice a year during the rainy seasons and feed on specific host plants. Predictive distribution models revealed that *B. alcinoe*, and *C. forda* are mostly found in tropical and sub-tropical regions in Africa. However, climate change could cause a slight decrease in their population by the year 2050. This information will guide conservation efforts and ensure sustainable use of edible saturniid caterpillars as food.

**Abstract:**

The promotion of edible insects, including saturniid caterpillars as potential food source is widely gaining momentum. They are adequately rich in nutrients such as proteins, amino acids, fatty acids, and micronutrients. Despite saturniids being a traditional food source with economic benefits, information on their diversity, host plants and their potential distribution in Africa are lacking, which this study seeks to address. Edible saturniids and their host plants were characterized using specific primers (LepF1/LepR1 and 3F_KIM_F/1R_KIM_R, respectively). Maximum entropy (MaxENT) and GARP (genetic algorithm for ruleset production) models were used to characterize the potential distribution of commonly consumed saturniids under current and future climate scenarios. Seven species of saturniids were recorded from 11 host plants in Kenya: *Gonimbrasia zambesina*, *Gonimbrasia krucki*, *Bunaea alcinoe*, *Gonimbrasia cocaulti*, *Gonimbrasia belina*, *Gynanisa nigra* and *Cirina forda*. Two morphotypes of *G. zambesina* and *B. alcinoe* were recorded. These saturniid caterpillars occur twice a year except for *G. cocaulti*. Predictive models revealed that tropical and subtropical regions were potentially suitable for *B. alcinoe* and *C. forda.* The information generated from this study would be important to guide conservation efforts and their sustainable utilization as food in Africa.

## 1. Introduction

The Food and Agriculture Organization of the United Nations has termed edible insects as one of the solutions to curb food insecurity [1]. Edible insects have been described as an alternative protein source [2] and recent studies have shown that they are a rich source of antioxidants [3] and are beneficial to the human gut microbiota [4]. Production of these insects is more sustainable compared to livestock since they require less land area [5], they offer a more efficient feed conversion [2] and emit less greenhouse gases [6]. Furthermore, edible insects require less water for mass production [7] and have the potential to be reared on bio-waste [8,9], which could in turn lower the cost of production.

Globally, more than 2 billion people consume over 2000 species of edible insects [2,10]. About 60% of edible insect species in Africa belong to the order Lepidoptera which includes edible saturniids [11]. Saturniids occur widely across Africa, USA, Australia and Asia [12,13,14]. Edible saturniids availability and consumption has been documented in West [15,16], Central [17] and southern Africa [18,19,20]. However, knowledge on the species diversity and edible saturniids consumed in East Africa is scarce. Most studies on edible saturniid caterpillars concentrated on western and southern Africa [21].

Edible saturniids are characterized by big larval forms with spines on the surface which pupate into cocoons [22] that are formed on the plants or leaf litters in the ground from which brightly colored moths emerge [12,13,14,23]. The most economically important saturniid that is consumed widely in southern Africa is the mopane worm, *Gonimbrasia belina* (Westwood). The larvae of *Go. belina* is an important food commodity among rural communities that live around the mopane woodland, i.e., the mopane belt across Angola, Zambia, Zimbabwe Mozambique, South Africa and Botswana [24]. They have been commercialized and contribute considerably to the rural economies; for instance, in Limpopo, South Africa, 63% of the harvested worms are sold in the local markets [20]. Another edible saturniid larvae in Africa with high commercial value is the pallid emperor moth or shea defoliator, *Cirina forda* (Westwood). Its larvae are considered an important food source for many rural communities in Zimbabwe, Nigeria, Togo, Ghana, Zambia, D.R. Congo, Central African Republic, and South Africa [16,25,26].

Edible saturniid species have been observed to inhabit different bioecological zones with considerable seasonal variability, as well as having high specificity and preference to various host plants. For example, in southern and eastern Africa, they are known to occur in large outbreaks in arid and savannah regions [12,14,27]. Edible saturniids can either be bivoltine or univoltine depending on the region [16,17,23,28,29,30]. Larvae of the mopane worm have been observed to feed specifically on *Colophospermum mopane* Kirk ex J. Léonard (Fabaceae) [31] while, *C. forda* show higher preference for the shea butter tree, *Vittelaria paradoxa* C.F. Gaertn (Sapotaceae) in West Africa [32,33].

Edible saturniid caterpillars are highly nutritious, providing vital vitamins, lipids and proteins and microelements to households, especially women and children [3,18,34,35,36]. Despite being highly nutritious, the diversity of edible saturniid caterpillars has not been studied in Kenya. Semi-wild rearing of edible saturniid caterpillars could promote proper land use management and forest conservation in an agroforestry setting [37]. The scarcity of information on edible saturniids and their distribution and host plants in Kenya has hampered the prospects of promoting their sustainable access and consumption as food among communities in Kenya. Knowledge on the host plants and the distribution of saturniids in Kenya may also promote their conservation in the ecosystem. The information on the host plants could open new opportunities for their mass production to ensure continuous supply. Therefore, the aim of the present study was to establish the diversity, distribution, and host plants of edible saturniids in Kenya. Further habitat suitability maps were also generated for selected saturniid species to assess their distribution under present and future climate change scenarios.

## 2. Materials and Methods

### 2.1. Study Area

The study was carried out between March 2017 and May 2019 across different agro-ecological zones [38,39] and altitudes in Kenya. The highland areas (1200–2000 m above sea level (meters above sea level, m.a.s.l.)) included Nakuru, Laikipia, Tharaka Nithi, Embu, Meru, and Nairobi counties, while the lowlands areas (0–750 m.a.s.l.) were represented by the counties of Makueni, Taita, Kwale and Kilifi. The middle altitude areas (750–1200 m.a.s.l.) included Homabay, Kitui, Kajiado and Machakos counties (Table S1). Some additional samples collected from Ibadan, Nigeria and Tutume, Botswana alone were included for comparison with samples collected in Kenya.

### 2.2. Sample Collection and Preparation

Saturniid larvae were sampled at random along motorable roads from 15 sites (Table 1) in Kenya. Mostly fourth and fifth instar stages were collected, placed in buckets with twigs from their host plant and transported to *icipe*, Nairobi, Kenya, for rearing and identification. Twigs of host plants were collected and pressed in a herbarium for identification. Sampling was done for five rainy seasons between March 2017 and May 2019, both long (March to May) and short rains (October–December), in Kenya.

Field-collected Saturniid larvae were reared at 12 h:12 h photoperiod at 25 °C in Perspex cages (50 × 50 × 50 cm) with nets on the sides for ventilation. Cages were protected from ants and crawling insects with traps containing water placed below its metallic stand. The saturniid larvae were fed on twigs of their respective host plant from the field. The twigs were placed in a container as bouquet with stem immersed in water fastened by wet cotton wool. The twigs were changed daily to keep them fresh. The larvae fed until they reached the pre-pupal stage, when they stopped feeding, reduced movement, and moved to the floor of the cage ready to burrow and pupate. The pre-pupae were placed on moist sterile sawdust in plastic trays and allowed to burrow into the sawdust to pupate as they burrow in soil in the wild. The saw dust was kept moist by sprinkling water daily. The trays with the pupae were placed in Perspex cages (50 cm × 50 cm × 50 cm) awaiting emergence of adult moths.

### 2.3. Morphological Identification

The adult moths were killed by placing them in a container and freezing them at −5 °C. They were stretched out, pinned, allowed to dry, and labeled before morphological identification. Identification was carried out using published keys [12] and crosschecked with reference voucher specimens at National Museums of Kenya (NMK) collection and pictures from available literature [40,41] by Mr. Alex Musyoki, Mr Ashikoye Okoko and Dr. Esther Kioko, NMK. Voucher specimens are deposited at the Biosystematics Unit, *icipe*. Host plants were identified at the Kenya Forest Research Institute (KEFRI) by an experienced plant taxonomist using available literature [42].

### 2.4. Molecular Identification

#### 2.4.1. Tissue Preparation, DNA Extraction and Quantification

Leaf samples of each host plant of saturniid caterpillar were carefully washed with tap water, rinsed with distilled water and dried with paper towel. They were then cut into 0.2 g of leaf sample small pieces with a sterile blade and placed in a 2 mL tube containing ceramic beads, lysis buffer PA1 (Bioline, London, UK) and RNAse A and crushed for 3 min in a Tissue lyser II (Qiagen, Germantown, MD, USA). Plant genomic DNA was extracted using Isolate II Plant DNA extraction Kit (Bioline, London, UK) as per the manufacturer’s instructions.

A leg of each adult moth and/or a portion of larvae collected were cut with a sterile blade and placed in a 2 mL tube. Insect genomic DNA was extracted using Isolate II genomic DNA extraction kit (Bioline, London, UK) as per the manufacturer’s protocol. The resultant DNA was eluted in 50 µL Elution buffer (Bioline, London, UK) and quantified using a NanoDrop 2000/2000 c spectrophotometer (Thermo Fisher Scientific, Wilmington, NC, USA). Insect and plant DNA samples were stored at −20 °C for further downstream processing.

#### 2.4.2. PCR for Insect Samples

For insect identification, PCR was conducted using general insect DNA barcoding LepF1/LepR1 primers (LEP F1-5′ ATTCAACCAATCATAAAGATATTGG 3′; LEP R1 5′ TAAACTTCTGGATGTCCAAAAAATCA 3′) [43]. Isolated insect DNA was amplified in 30 µL PCR mix containing 17.025 µL PCR water, 6 µL My Taq Buffer (Bioline, London, UK), (5 mM dNTPs, 15 mM MgCl2, stabilizers and enhancers), 1.5 µL of each primer, 0.6 µL of 25 mM MgCl2 (Thermo Fisher Scientific, Waltham, MA, USA), 0.375 µL 1 unit My Taq DNA polymerase (Bioline, London, UK) and 15 ng/L of DNA template. The reaction was set up in a Mastercycler Nexus Gradient thermocycler (Thermo Fisher Scientific, Waltham, MA, USA) using conditions as follows: initial denaturation at 95 °C for 2 min followed by 40 cycles of denaturation at 95 °C for 30 s, annealing at 52 °C for 40 s and primer elongation at 72 °C for 1 min. The final extension step lasted for 10 min at 72 °C.

#### 2.4.3. PCR for Plant Samples

General plant primers 3F_KIM_F/1R_KIM_R (3F_KIM_F 5′ CGTACAGTACTTTTGTGTTTACGAG 3′; 1R_KIM_R5′ ACCCAGTCCATCTGGAAATCTTGGTTC 3′) were used to amplify a 900 bp region of the matK gene for the identification of host plants. Protocols for PCR of plant samples were similar to the PCR protocol for insect samples (Section 2.4.2), except the annealing step which was done at 49 °C for 45 s.

#### 2.4.4. Agarose Gel Electrophoresis, PCR Product Purification and Sequencing

Resolution of the PCR product was done with 1% agarose gel stained with ethidium bromide (10 mg/mL) at 80 volts for 1 h (Bio-Rad model 200/2-0 power supply, Bio-Rad laboratories Inc., Hercules, CA, USA). DNA bands were visualized using an ultraviolet transilluminator and photographed using the KETA GL imaging system software (Wealtec Corp., Sparks, NV, USA). The resultant PCR products for both the insects and the host plants were purified using QIAquick PCR purification kit (Qiagen, Hilden, Germany) and quantified with a NanoDrop 2000/2000 c spectrophotometer (Thermo Fisher Scientific, Wilmington, NC, USA) before being sent for bidirectional sequencing at Macrogen Inc. (Amsterdam, The Netherlands).

#### 2.4.5. Sequence Analysis

Both plant and insect sequences were assembled and edited using Bioedit software v. 7.0.5.2 [44]. A consensus sequence generated from both the forward and reverse strand was queried on Basic Local Alignment Search Tool (BLAST) [45] and Barcode Of Life Data system (BOLD) [46] to determine similarity with sequences in the database. The default Species level barcode records were used. The top published hit on Bold was used for identification. Multiple sequence alignments were created on Clustal W [47]. Pairwise distances were generated using using Mega X [48]. Sequences were submitted to the GenBank (https://www.ncbi.nlm.nih.gov/WebSub/) (accessed on 10 March 2020) (see Table S2).

### 2.5. Distribution Modelling

Two species of edible saturniids were selected for distribution modeling, i.e., *B. alcinoe* and *C. forda*, because of their popularity and economic importance in sub-Saharan Africa (SSA). *Cirina forda* and *B. alcinoe* are widely consumed [11,17,49,50,51] and traded [16,52,53,54] in western, central, eastern, and southern Africa.

#### 2.5.1. Occurrence Data

**Figure 1:**
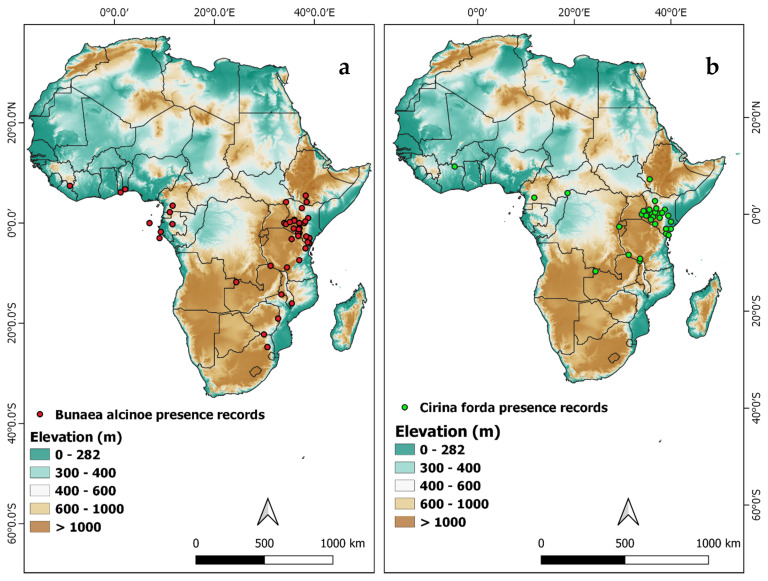
Geographical distribution records of (**a**) *Bunaea alcinoe* and (**b**) *Cirina forda* in Africa.

The occurrence data (species name, GPS co-ordinates) for the two species was collected during field surveys in Kenya and from the Global Biodiversity Information Facility (GBIF). A total of 96 points (Figure 1a) were acquired (59 from field surveys and 37 from GBIF) for *B. alcinoe*, while the *C. forda* dataset comprised 70 points (57 from field surveys and 13 from GBIF) (Figure 1b).

#### 2.5.2. Environmental Variables

Nineteen bioclimatic variables downloaded from WorldClim (www.worldclim.org) (accessed on 15 April 2021) were considered for the study. We obtained current climate data for the period 1970–2000 at 30 arc seconds longitude/latitude degree spatial resolution (approximately 1 km at the equator). For future analysis, the downscaled and calibrated horizon 2050 IPPC (CMIP5) of climate projection bioclimatic variables were extracted from HadGEM2-ES global climate model (GCM) representing representative concentration pathways (RCP8.5) for future climate scenarios set by the intergovernmental panel on climate change (IPCC) [53]. RCP8.5 scenario predicts the mean global temperature increase projections of up to 3.7 °C. A collinearity test was conducted on the 19 bioclimatic variables to reduce collinearity between variables, to avoid overfitting of the model and variable inflation [54]. The variance inflation factor (VIF) test was used to assess the correlation between variables. The “vifcor” function in R software version 3.0.1 was used to run the VIF test [55]. Six bioclimatic variables, namely Bio2 (mean diurnal temperature range), Bio3 (Isothermality), Bio5 (max temp of warmest month), Bio13 (precipitation of wettest month), Bio15 (precipitation seasonality), and Bio19 (precipitation of coldest quarter) were selected for the *B. alcinoe* species analysis, while seven bioclimatic variables namely, Bio2, Bio4 (temperature seasonality), Bio8 (mean temperature of wettest quarter), Bio13, Bio15, Bio18 (precipitation of warmest quarter), and Bio19 were selected for the *C. forda* species analysis using a cutoff of |r2| > 0.7. Aside from the spatial correlation, the ecological relevance of the variables was also considered.

#### 2.5.3. Model Calibration and Accuracy Assessment

Ecological niches of the two species were modeled using Maximum Entropy (MaxEnt) in the MaxEnt tool package version 3.4.1k which performs well for modeling presence only data [55]. The ENMEval package in R software was used to determine the required parameter settings to be used in Maxent software for the optimum tuning of the models [56]. Following the parameter settings from ENMEvaluate, three features (linear, quadratic and hinge) were utilized with a regularization multiplier of 3. The model calibration created the optimal models for the two saturniid species. The models were replicated 3 times using cross-validation method and an ensemble of the three probability outputs were used to determine the optimum suitability and performance of the models. Seventy percent of the presence records were utilized to train the model while 30% of the points were used to validate the performance of the model. The comparative relevance of each environmental variable for the models of *C. forda* and *B. alcinoe* was evaluated using the overall percentage contribution, area under the curve (AUC), and the Jackknife test. AUC values of 0 indicate impossible occurrence area while 1 indicates optimal occurrence area. The ROC method has shown to be effective in evaluating model performance and being independent of prevalence [57,58]. Outputs of the models highlighting the intensity and extent of habitat suitability of the two species were mapped with values ranging from 0 (unsuitable) to 1 (optimum). Suitability levels were grouped into five categories as follows: very low (0–0.1), low (0.1–0.3), moderate (0.3–0.5), high (0.5–0.7), and very high (0.7–1).

## 3. Results

### 3.1. Morphological Identification of Edible Saturniids

Seven species of Saturniidae were identified in Kenya. They include *B. alcinoe*, *C. forda*, *Gonimbrasia zambesina* (Walker), *Go. cocaulti* Darge and Terral, *Go. belina* Westwood, *Go. krucki* (Hering) and *Gynanisa nigra* Bouvier. Dead larval stages of *Gonimbrasia belina* collected from Botswana and a sample of *B. alcinoe* collected from Nigeria were included for comparison (Table 1).

#### 3.1.1. *Bunaea alcinoe* 

**Figure 2:**
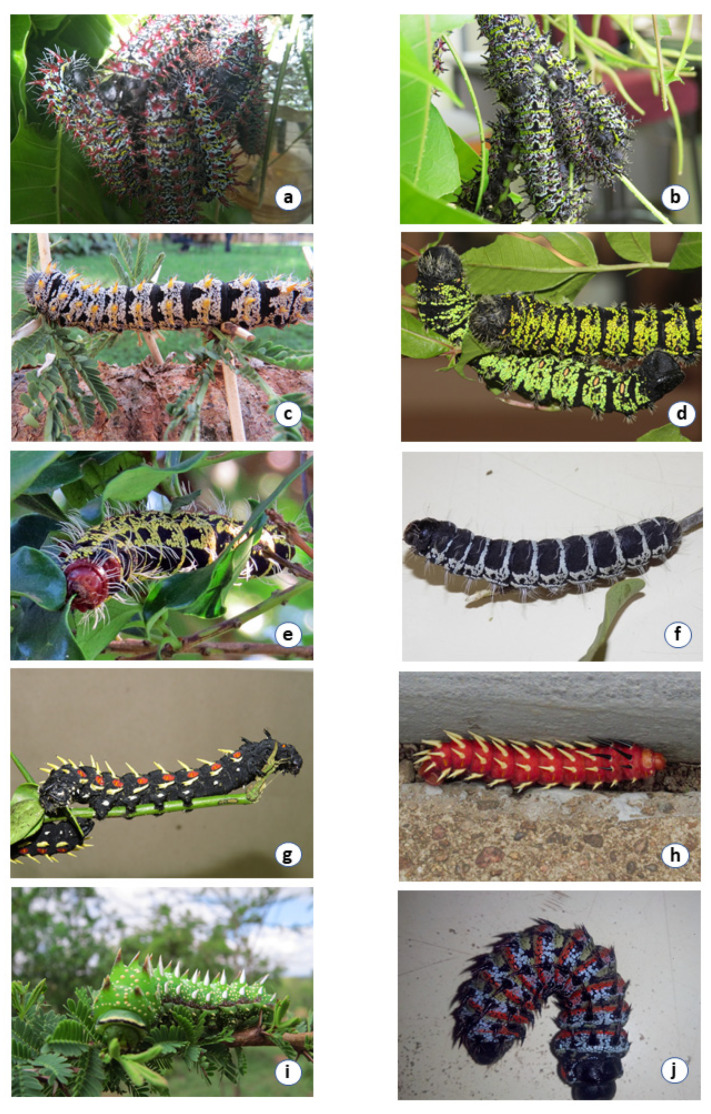
Pictures of saturniid larvae. (**a**) *Gonimbrasia zambesina* larvae with red spines, (**b**) *Gonimbrasia zambesina* larvae with black spines, (**c**) *Gonimbrasia cocaulti*, (**d**) two color forms of *Gonimbrasia krucki* larvae; black with green speckles and black with yellow speckles, (**e**) *Cirina forda* larva black in color with yellow spots, (**f**) *Cirina forda* larva black in color with white spots, (**g**) *Bunaea alcinoe* black form observed in East Africa, (**h**) *Bunaea alcinoe* red form observed in West Africa, (**i**) *Gynanisa nigra* larvae, (**j**) *Gonimbrasia belina* collected in Kenya.

**Figure 3:**
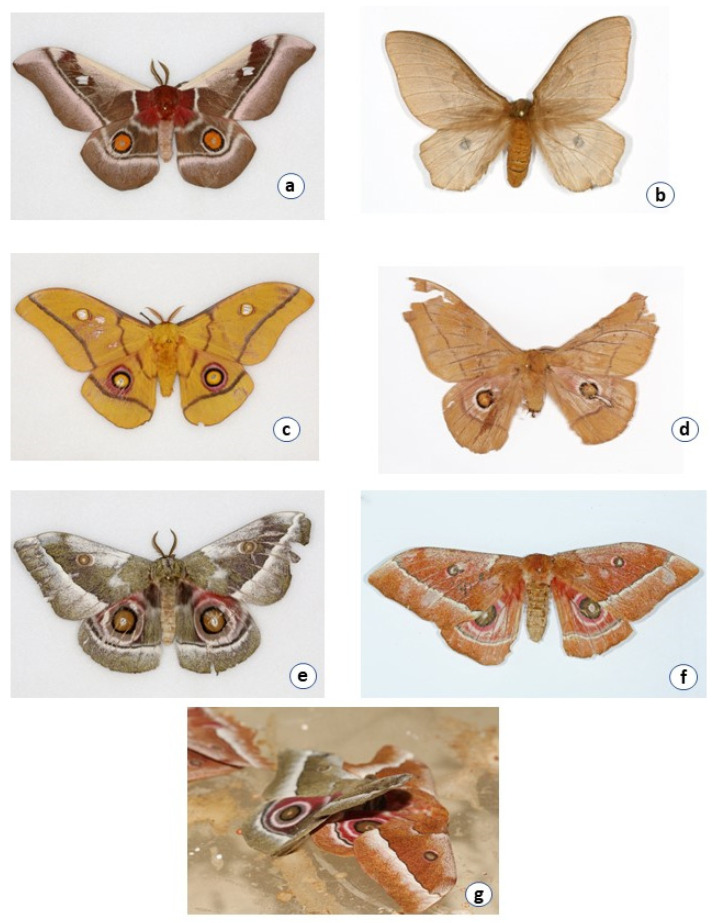
Pictures of saturniid moths. (**a**) *Bunaea alcinoe*, (**b**) *Cirina forda*, (**c**) *Gonimbrasia krucki*, (**d**) *Gonimbrasia belina*, (**e**) green form of *Gonimbrasia zambesina*, (**f**) brown form of *Gonimbrasia zambesina*, (**g**) mating pair of brown and green form of G. *zambesina*.

Larvae are black with orange spots on the spiracles along the sides of the body. One *B. alcinoe* larva collected from Nigeria is red in color. Larvae of both color forms of *B. alcinoe* have white/yellow spines (Figure 2). *Bunaea alcinoe* moths are dark brown in color with a large glass spot on the forewing (Table 1). The hind wing has an orange eyespot ringed with black followed by white (Figure 3).

#### 3.1.2. *Cirina forda* 

Larvae are black with yellow bands and white hairy spines. *Cirina forda* moths are smaller than the other moths. *Cirina forda* larvae in the same colony also presented in two color forms. Some had a black body with yellow bands while others had a black body with white bands (Figure 2). It is light brown in color with a small black eyespot on the hindwing (Table 1, Figure 2 and Figure 3).

#### 3.1.3. *Gonimbrasia cocaulti* 

Larvae appear black with whitish speckles and yellow spines. *Cirina forda* moths are smaller than the other moths. It is light brown in color with a small black eyespot on the hindwing (Figure 3). *Gonimbrasia cocaulti* moths have a brownish ground color with eyespots on both the forewing and hindwing. The eyespot on the hindwing is white surrounded by reddish, black, and white rings while the one on the forewing is whitish circled by a brownish and white ring (Table 1, Figure 2 and Figure 3).

#### 3.1.4. *Gonimbrasia krucki* 

Larvae are black with greenish-yellow speckles and black thick spines. *Gonimbrasia krucki* presented two color forms of their larvae. Larvae produced from the same egg clutch developed into forms that had a black body with either yellow or green speckles (Figure 2). *Gonimbrasia krucki* moths have a yellow ground color with defined eyespots on both the forewing and the hindwing. Both eyespots are yellow in color ringed with black, pink, and red (Figure 3).

#### 3.1.5. *Gonimbrasia belina* 

Larvae are red, grey, and green with black spines. *Gonimbrasia belina* moths are reddish brown in color with a brown eyespot on the hind wing circled by black and white rings. It has a small glass spot on the forewing. The front part of the hindwing is reddish in color (Figure 2 and Figure 3).

#### 3.1.6. *Gonimbrasia zambesina* 

Larvae are black with yellow and grey speckles while some have black spines and others red spines (Table 1, Figure 2)*. Gonimbrasia zambesina* moths occur in two color forms, green and brown. The green form is reddish purplish on the forward part of the hindwing. The eyespot on the hindwing is greenish yellow in the middle, circled by a black ring followed by greenish-yellow and white. The brown form has a yellowish-brown eyespot on the hind wing with black, pink, and whitish rings (Figure 3). The brown form is not available in the NMK collection. The dichotomous key [12] reported the specimen as *Go. said* (Oberthuer). However, the author expressed uncertainty and suggested that it could be a form of *G. zambesina*. The green forms were collected from Kilifi, Embu and Kwale, while a mixture of the green and brown forms was collected from Makuyu in Murang’a County (all Kenyan sites). The green moths from Kwale and Kilifi produced larvae that were black with grey and yellow speckles and black spines (Figure 2). Green moths from Embu laid eggs that hatched into larvae that were black with grey and yellow speckles and with red spines. The green and brown moths were also observed to mate with each other. The brown and green moths collected in Makuyu mated among themselves (green and green/green and brown/brown and brown) to produce black larvae with grey and yellow speckles and with red spines (Figure 3).

#### 3.1.7. *Gynanisa nigra* 

Larvae of *Gy. nigra* are green with white speckles and white spines (Figure 2). We could not get adult moth from field collected *Gy. nigra* due to extensive parasitism.

### 3.2. Molecular Identification

All the saturniid species showed 98.22–100% similarity to sequences in BOLD. *Gonimbrasia belina* collected in Kenya and *Gy. nigra* were 100% similar to sequence GBMNC60703-20 (*Go. belina*; BIN-BOLD:AAB6786) and GBMNC60687-20 (*Gy. Nigra*; BIN- BOLD:AED6623), respectively. *Gonimbrasia zambesina* sequences were 99.09–100% similar to SAPBA773-07 (*Go. Zambesina*; BIN-BOLD:AAD1339)) while *Go. krucki* was 98.22–100% similar to SAPBA635-07 (*Go. Krucki*; BIN-BOLD:AAD8374)). *Cirina forda* had 99.38–99.85% similarity to SATWA281-07 (*C. forda*; BIN-BOLD:AAB6982) and STBOB620-08 (*C. forda*; BIN-BOLD:AAB6982). *Gonimbrasia cocaulti* sequences were >98% similar to SPBIS152-09 (*Go. Cocaulti*; BIN-BOLD:AEH8028). *Bunaea alcinoe* sequences from samples collected in Kenya were 98.78–100% similar to LSAFR2238-12 from South Africa (*B. alcinoe*; BIN-BOLD:AAA6757) (Table 2).

*Gynanisawestwoodi* and *G. belina* collected from Botswana were both >99% similar to STBOA580-07 (BIN-BOLD: ABY462) and SATWA003-06 (BIN-BOLD: AAB6786), respectively. *Bunaea alcinoe* sequences from sample collected in Nigeria was 100% similar to SATWA891-07 (*B. alcinoe*; BIN-BOLD: AAA6756) (Table 2).

*Gonimbrasia krucki* and *B. alcinoe*-Nigeria species had a 100% similarity to sequences from BOLD database. *Gonimbrasia zambesina* had a within species pairwise distance range of 0–0.61 and a range of 0–0.91 between species and BOLD sequence SAPBA773-07 (BOLD:AAD1339) (Table 3). *Gonimbrasia belina* collected in Botswana and *Gynanisa westwoodi* had a 100% similarity within species, while they had a pairwise distance range of 0.15–0.16 and 0.3 with BOLD sequences SATWA003-06 (BOLD:AAB6786) and STBOA580-07 (BOLD:ABY4629), respectively (Table 3). *Gonimbrasia belina* collected in Kenya was 100% similar to the BOLD sequence, while *G. nigra* had a pairwise distance of 0.61. *Cirina forda* showed a pairwise distance range of 0.3–2.13 within species and 0.46–2.13 between species and BOLD sequence SATWA281-07 (BOLD:AAB6982) (Table 3).

#### Molecular Differences among the Color Forms of *Gonimbrasia zambesina* and *Bunaea alcinoe*

**Figure 4:**
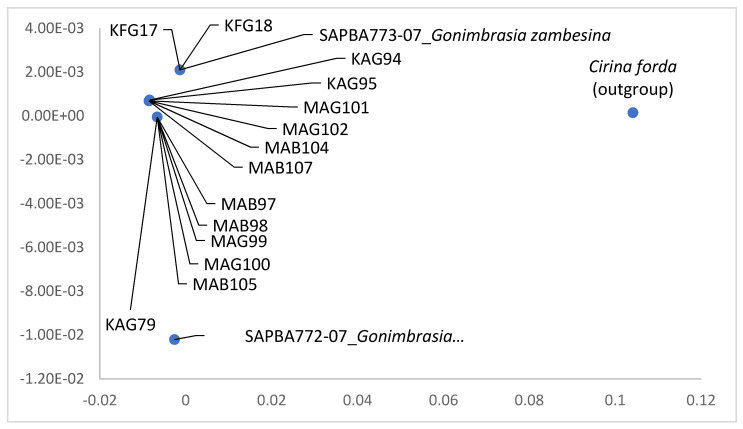
Principle coordinate analysis of COI gene of different morphotypes of *Gonimbrasia zambesina* from different locations in Kenya. The first two letters indicate the collection sites (MA = Makuyu, KA = Kambiti, and KF = Kilifi). The last letter indicates the color of the adult moth (G = green and B = brown). The number indicates sample ID. SAPBA772-07 and SAPBA773-07 are BOLD reference accession numbers in BOLD BIN cluster BOLD:AAD1339.

Although *Go. zambesina* larvae and moths depicted different color forms morphologically, they identified as the same species using molecular characteristics. The genetic distance between all the *Go. zambesina* samples and the reference *Go. zambesina* sequence from BOLD (SAPBA773-07; BIN BOLD:AAD1339) was 0–0.61% while the genetic distance between all the samples and *Go. zambesina* sequence from BOLD (SAPBA772-07) was 1.07–1.23%. The genetic distance between the samples was 0–0.61%. All the samples included in the analysis had 658 bp (Figure 4).

**Figure 5:**
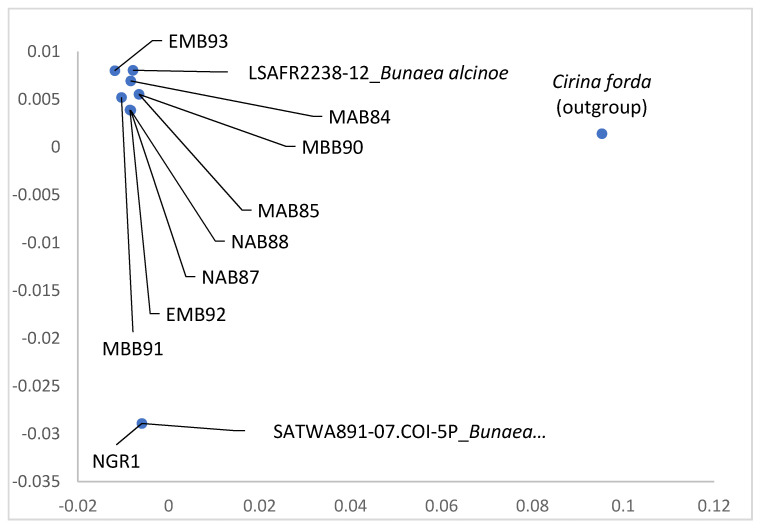
The principle coordinate analysis of COI gene of morphotypes of *Bunaea alcinoe* from Kenya and Nigeria. The first two letters indicate the collection sites (NG = Nigeria, MB = Mbita, EM = Embu, NA = Nanyuki and MA = Matuu). The last letter depicts the color of the larvae (R = red and B = black). The number indicates sample ID. SATWA891-07.COI-5P (BIN BOLD:AAA6756) and LSAFR2238-12 (BIN BOLD:AAA6757) are BOLD reference accession numbers.

The morphological difference of the two larvae color forms of *B. alcinoe* was also supported by molecular characterization. The red form with white spines (Nigeria-1) was 100% similar to SATWA891-07-COI-5P (*B. alcinoe;* BIN BOLD:AAA6756) from Burkina Faso. However, the same BOLD sequence had a 3.28–3.76% genetic distance from all the other black larvae forms of *B. alcinoe* collected from Kenya (see Table S4. All the other black forms with white spines from Kenya showed a genetic distance of 0.61–0.76% from LSAFR2238-12 (*B. alcinoe*; BIN BOLD:AAA6757) from South Africa. The same BOLD sequence had a genetic distance of 3.76% from the red color form collected from Nigeria. All the black forms clustered together with LSAFR2238-12 (*B. alcinoe*) while the red form clustered with SATWA891-07-COI-5P (*B. alcinoe*). All the samples included in the analysis had 658 bp (Figure 5).

### 3.3. Distribution and Seasonality of Edible Saturniids in Kenya

The distribution of the edible saturniids in the various Counties in Kenyan is presented in Table S1. *Gonimbrasia zambesina*, *C. forda*, *Go. krucki*, and *B. alcinoe* were bivoltine occurring between April–June and October–December, reflecting the major and minor rainy seasons in the region. On the other hand, *Go. cocaulti* was univoltine and occurred only during the April–June season (Table 4). The distribution of these saturniids was attributed to the availability of their host plants. The most widespread saturniid was *B. alcinoe* while the least widespread were *Go. belina* and *Go. krucki* which were only found in Kwale and Nairobi, respectively (Table 4).

### 3.4. Habitat Suitability and Probability Distribution

#### 3.4.1. Area under Curve (AUC) Values

All the models using the current and future (RCP8.5:2050) showed a balance between goodness-of-fit and complexity (AUC > 0.80), for all the test and training datasets (Table 5). This demonstrates that our models showed good predictive performance.

#### 3.4.2. Visualization of Habitat Suitability under Current and Future Climatic Conditions

**Figure 6:**
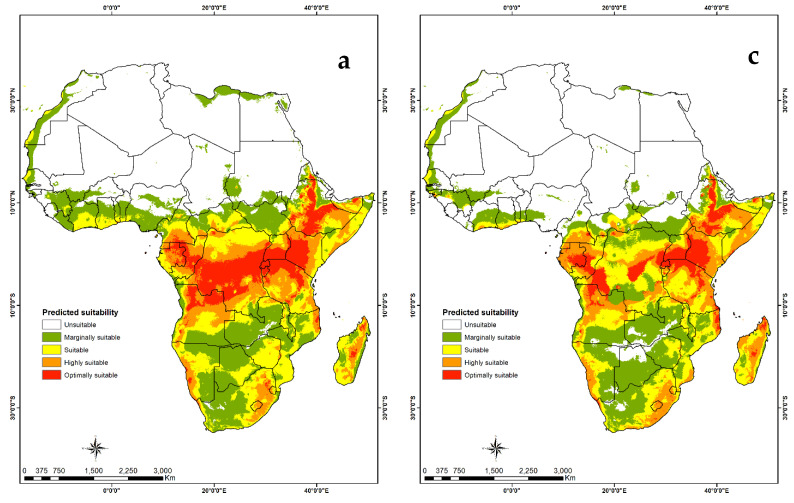

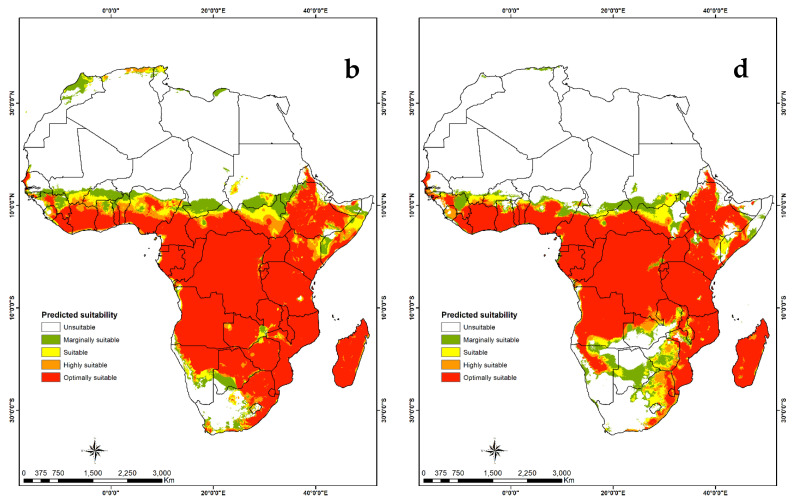
Predictive suitability map of the geographic distribution of *Bunaea alcinoe*. (**a**) Suitability map generated by the MaxEnt algorithm under current climate scenario; (**b**) suitability map generated by the GARP algorithm under current climatic scenario; (**c**) suitability map generated by MaxEnt algorithm under future climatic scenarios and (**d**) suitability map generated by the GARP algorithm under future climatic scenarios.

**Figure 7:**
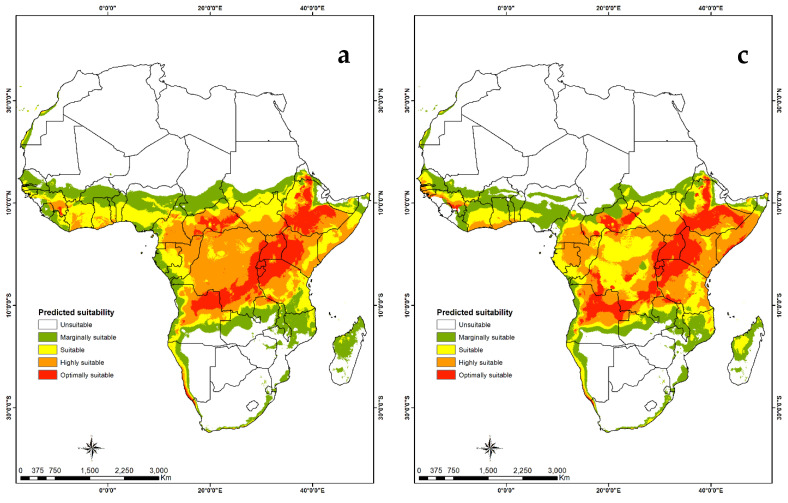

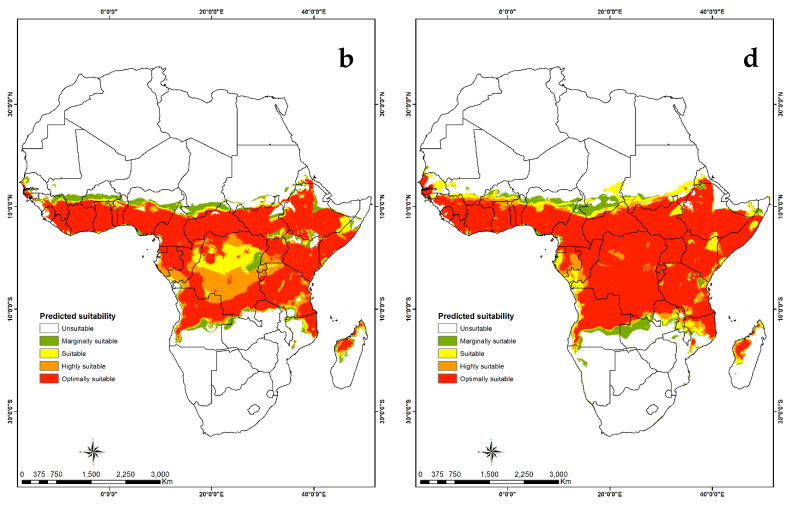
Predictive suitability map of the geographic distribution of *Cirina forda*. (**a**) Suitability map generated by the MaxEnt algorithm under current climate scenario; (**b**) suitability map generated by the GARP algorithm under current climatic scenario; (**c**) suitability map generated by MaxEnt algorithm under future climatic scenarios and (**d**) suitability map generated by the GARP algorithm under future climatic scenarios.

Habitat suitability maps show that the tropics are optimal for both *B. alcinoe* (Figure 6) and *C. forda* (Figure 7). Parts of the subtropical region in southern Africa are marginally suitable for *B. alcinoe*. For both MaxENT and GARP maps, a slight reduction in habitat suitability for both saturniids is predicted in future climate scenarios. Northern Africa is unsuitable in both present and future scenarios for *B. alcinoe* and *C. forda.*

### 3.5. Host Plants of Edible Saturniids in Kenya

The caterpillars were observed to show very distinct host plant specificity across the various sites surveyed. We observed that *Go. cocaulti* and *B. alcinoe* mainly feed on *Vachellia nilotica* (L.) P.J.H. Hurter and Mabb (Fabaceae), while *B. alcinoe* was feeding on *Balanites aegyptiaca* Linn (Zygophyllaceae) and *Balanites glabra* Mildbr. and Schltr. (Zygophyllaceae). *Gonimbrasia cocaulti* also fed on *Vachellia tortilis* (Forssk.) Galasso and Banfi (Fabaceae), while *Go. zambesina* and *Go. belina* on *Anacardium occidentale* Lin (Anacardiaceae) and *Go. zambesina* as well on *Mangifera indica* L. (Anacardiaceae). *Gonimbrasia krucki* was only observed feeding on *Schinus terebinthifolia* Raddi and *Schinus molle* L. (Anacardiaceae) while *C. forda* seemed to have a broader host range with *Euclea divinorum*, *Acacia mearrnsii* De Wild (Fabaceae) and *Manilkara sulcata* Engl. (Sapotaceae) (Table 6).

In terms of genetic analyses, *E. divinorum*, *A. occidentale*, *M. indica*, *Balanites* sp., *V. tortilis*, *Manilkara* sp., *A. mearnsii*, *S. terebinthifolia* and *V. nilotica* had a 97.06–100% similarity to GenBank sequences (Table 7).

## 4. Discussion

This study has documented seven species of edible saturniids in Kenya. Three saturniid species, i.e., *C. forda*, *Go. zambesina* and *B. alcinoe*, are consumed in Kenya, mainly along the coastal belt along the Giriama community. Moreover, *C. forda* is widely consumed in West, Central and southern Africa [15,16,33,51]. *Bunaea alcinoe* is also a popular edible insect in West and Central Africa, for instance in countries like DR Congo, Cameroon and Nigeria [59,60,61], while *Go. zambesina* is highly popular in southern and Central Africa [17,59,62]. *Gonimbrasia krucki* is widely consumed in DR Congo [17], but not in Kenya. For *Go. cocaulti*, no records of human consumption are available from Kenya or elsewhere in Africa. However, due to the similarity of *Go. cocaulti* larva to that *Go. belina,* it is likely misidentified, given that they have been observed in consignments of mopane caterpillar in the UK [63].

*Bunaea alcinoe,* though black in colour and commonly found in Kenya, its genetic configuration is different from the red forms collected in Nigeria [64]. In Nigeria and DR Congo, both color forms have been reported feeding on the same host plant [17,64], yet this is the first time their genetic difference has been assessed. Further detailed studies relating the morphological and molecular differences, mating compatibility between color forms of *B. alcinoe* can shed light on their taxonomic status.

The sampled *G. zambesina* moths also exhibited two color forms and juveniles of the green adults carried black spines, as previously reported [12] and in http://www.africanmoths.com/ (accessed on 26 September 2019) [41]. The brown moths emerged from red spined larvae, which had been previously described as *G. said* [12], but inconclusively. Identification of edible saturniid species is important for the purpose of conservation [37,65], maintenance of quality in production [63] as well as mainstreaming consumption of the caterpillars.

We observed a bivoltine lifecycle in *C. forda*, with larvae occurring in April–June and October–December. In contrast, *C. forda* has been recorded as univoltine in Togo and Nigeria with larval occurrence between July and September [16,29] and in DR Congo with larvae appearing between November and January [17]. In all these cases, the occurrence of *C. forda* larvae coincides with the rainy seasons. For, *B. alcinoe* we noted a bivoltine lifecycle with larval appearance between April–May and October–December. In contrast, the same species in DR Congo is univoltine and occurs between October and May [17]. Understanding the temporal distribution of edible saturniids informs the need for mass production to ensure a continuous source throughout the year.

The model for habitat suitability of *B. alcinoe* and *C. forda* demonstrates that the two species thrive well within the tropical regions of Africa. However, *B alcinoe* spreads slightly into the subtropics, specifically in southern Africa. The model concurs with previous reports of availability and consumption of the two edible saturniids in southern, central and western Africa [11,16,17,28,33,51,59,66]. However, the availability of *C. forda* in the southern African region is not concurrent with previous reports [14,23] which recorded a wide distribution in the southern Africa region. This could be due to the limited data on the presence of the two saturniid species in the GBIF database which was used in this study. Future predictions for both species show a slight reduction in habitat suitability, stressing the need to conserve edible saturniid species’ habitats.

The saturniids identified fed on specific host plants and consequently their availability depends on the occurrence of these host plants. We found *B. alcinoe* feeding on *B. aegyptiaca* and *B. glabra* similar to reports in Nigeria [63]. However, other research suggests a wide range of host plants, e.g., for DR Congo with *Sarcocephalus latifolius* (JE Sm.) EA Bruce (Rubiaceae), *Acacia auriculiformis* A. Cunn. ex Benth. (Fabaceae), *Dacryodes edulis* (G. Don) H.J.Lam. (Burseraceae), *Crossopteryx febrifuga* (Afzel. ex G.Don) Benth. (Rubiaceae) and *Anthocleista schweinfurthii* Gilg (Loganiaceae) [13], and in Nigeria on *Holarrhena floribunda* (G. Don) Durand and Schinz (Apocynaceae), *Ekebergia sengalensis* A Juss. (Meliaceae), *Fragraea fragrans* Roxb. (Gentianaceae), *Cleistopholis patens* (Benth.) Engl. and Diels (Annonaceae) and *Spondias mombin* L. (Anacardiaceae) [63]. In our study in Kenya we observed, for the first time, larvae of *C. forda* feeding on *E. divinorum*, *A. mearnsii* and *Manilkara sulcate*, while in DR Congo it feeds mainly on *Crossopteryx febrifuga* [17]. In West Africa, *C. forda* is confined on the shea butter tree, *Vittelaria paradoxa* [16,29,33], whereas in southern Africa host plants include *Burkea africana* Hook (Fabaceae) and *Albizia versicolor* Welw. ex Oliv. (Fabaceae) [23].

We collected *Go. zambesina* from mango and cashew nut trees in Kenya, corroborating earlier findings [12]. Cashew nut and mango trees are important commercial trees whose nuts and fruits, respectively, are widely consumed. *Gonimbrasia zambesina* is sometimes considered a pest of mango trees [67]. Spraying of mango trees to curb pests may pose a threat to *Go. zambesina* larvae feeding on the leaves. 

Apart from being host plants for edible insects, most of these plants have other uses in communities in Kenya and beyond. For example, *E. divinorum* is utilized by the Maasai in Kenya as firewood, their stem cuttings are used as toothbrushes and their fruits are edible [68]. Marakwets from the Rift Valley region in Kenya use *E. divinorum* as anti-venom [69] while the Luo from western Kenya use it to treat venereal diseases [70]. Maasai also use *V. tortilis* and *V. nilotica* for firewood [71] while the Marakwet employ them for treating abdominal pains [69]. *Balanites* spp. are used to treat coughs [70], and finally *A. mearnsii* is often planted for firewood, timber, apiculture and a source of tanning dyes, and trees are also used for shade, nitrogen fixation and controlling soil erosion [72]. Such traditional knowledge can be used to encourage communities to conserve these plants and hence protect habitats for edible saturniid species.

## 5. Conclusions

We successfully documented seven species of saturniids in Kenya, among which three are consumed. The identity of these species was confirmed both at molecular and morphological level. Their distribution, seasonality and host plants were also established. We emphasize the importance of combining molecular barcoding, morphological identification, phenology, and ecology studies in identification of edible saturniid species. Potential habitats under current and future climate scenarios of two edible saturniid species, *B. alcinoe* and *C. forda*, were mapped. This information may help in implementing conservation measures for edible saturniids and their host plants. Due to their seasonal occurrence, further research is required on prospects for mass production to ensure a continuous supply and to prevent overharvesting from the wild forest for enhance sustainability. Moreover, potential economic benefits of edible saturniids for local communities in East Africa need to be quantified and their value chains established.

## Figures and Tables

**Table 1:** Morphological characteristics of saturniids collected in Kenya.

Species	Characteristics			
	Moth Color	Hindwing Eyespot Description (Innermost to Outermost Color)	Mature Larvae Color	5th Instar Spine Color
*Gonimbrasia zambesina*	Green	Greenish-yellow center, black, greenish-yellow, white rings	Black with grey and yellow speckles	Black or red
*Gonimbrasia zambesina*	Brown	Yellowish brown center, black, pink, white rings	Black with grey and yellow speckles	Red
*Gonimbrasia cocaulti*	Brown	White center, reddish, black and white rings	Black with whitish speckles	Yellow
*Gonimbrasia krucki*	Yellow	Yellow center, black, pink, red rings	Black with greenish-yellow speckles and orange spots on spiracles	Black
*Cirina forda*	Light Brown	Small with a black ring	Black with yellow bands	White
*Gonimbrasia belina*	Reddish-Brown	Brown center, black, white rings	Black with red, grey and green speckles	Black
*Bunaea alcinoe*	Dark brown	Orange center, black, white rings	Black with orange spots on spiracles	White/yellow
*Bunaea alcinoe*	-	-	Red	White
*Gynanisa nigra*	-	-	Green with white speckles	White
*Gonimbrasia belina*	-	-	Black with red, grey and greenish speckles	Black

‘-‘ Means moths were not collected.

**Table 2:** Identities of saturniids based on similarities with sequences from BOLD.

Collection Site	Sample Code	Species	% Similarity to BOLD Sequences	Sequence ID of Similar Sequences-Top Published Hit with Default BOLD Query (Collection Site)
Mwingi, Kenya	S30	*Go. cocaulti*	98.88	SPBIS152-09 (Kenya)
Taita, Kenya	S34	*Go. cocaulti*	98.88	SPBIS152-09 (Kenya)
Matuu, Kenya	S22	*Go. cocaulti*	98.72	SPBIS152-09 (Kenya)
Taita, Kenya	S32	*Go. cocaulti*	98.87	SPBIS152-09 (Kenya)
Taita, Kenya	S33	*Go. cocaulti*	98.88	SPBIS152-09 (Kenya)
Matuu, Kenya	S28	*Go. cocaulti*	98.72	SPBIS152-09 (Kenya)
Muhaka, Kenya	S48	*Go. belina*	100	GBMNC60703-20 (Kenya)
Ibadan, Nigeria	Nigeria-1	*B. alcinoe*	100	SATWA891-07 (Burkina-Faso)
Matuu, Kenya	S84	*B. alcinoe*	99.24	LSAFR2238-12 (South Africa)
Matuu, Kenya	S85	*B. alcinoe*	99.39	LSAFR2238-12 (South Africa)
Nanyuki, Kenya	S87	*B. alcinoe*	99.24	LSAFR2238-12 (South Africa)
Nanyuki, Kenya	S88	*B. alcinoe*	99.24	LSAFR2238-12 (South Africa)
Mbita, Kenya	S90	*B. alcinoe*	99.39	LSAFR2238-12 (South Africa)
Mbita, Kenya	S91	*B. alcinoe*	99.08	LSAFR2238-12 (South Africa)
Embu, Kenya	S92	*B. alcinoe*	99.23	LSAFR2238-12 (South Africa)
Embu, Kenya	S93	*B. alcinoe*	98.78	LSAFR2238-12 (South Africa)
Nairobi, Kenya	S2	*Go. krucki*	100	SAPBA635-07 (Kenya)
Nairobi, Kenya	S3	*Go. krucki*	100	SAPBA635-07 (Kenya)
Mbita, Kenya	S6	*C. forda*	99.54	SATWA281-07 (Cameroon)
Mbita, Kenya	S7	*C. forda*	99.54	SATWA281-07 (Cameroon)
Kilifi, Kenya	S55	*C. forda*	99.41	STBOB620-08 (Malawi)
Ngong, Kenya	2CF	*C. forda*	99.38	STBOB620-08 (Malawi)
Ngong, Kenya	5CF	*C. forda*	99.69	STBOB620-08 (Malawi)
Gilgil, Kenya	S54	*C. forda*	99.85	STBOB620-08 (Malawi)
Kilifi, Kenya	S17	*Go. zambesina*	100	SAPBA773-07 (Kenya)
Kilifi, Kenya	S18	*Go. zambesina*	100	SAPBA773-07 (Kenya)
Kambiti, Kenya	S79	*Go. zambesina*	99.54	SAPBA773-07 (Kenya)
Kambiti, Kenya	S94	*Go. zambesina*	99.39	SAPBA773-07 (Kenya)
Embu, Kenya	S95	*Go. zambesina*	99.39	SAPBA773-07 (Kenya)
Embu, Kenya	S96	*Go. zambesina*	99.58	SAPBA773-07 (Kenya)
Makuyu, Kenya	S97 Brown	*Go. zambesina*	99.54	SAPBA773-07 (Kenya)
Makuyu, Kenya	S98 Brown	*Go. zambesina*	99.54	SAPBA773-07 (Kenya)
Makuyu, Kenya	S99 Green	*Go. zambesina*	99.54	SAPBA773-07 (Kenya)
Makuyu, Kenya	S100 Green	*Go. zambesina*	99.09	SAPBA773-07 (Kenya)
Makuyu, Kenya	S101 Green	*Go. zambesina*	99.39	SAPBA773-07 (Kenya)
Makuyu, Kenya	S102 Green	*Go. zambesina*	99.39	SAPBA773-07 (Kenya)
Makuyu, Kenya	S104 Brown	*Go. zambesina*	99.39	SAPBA773-07 (Kenya)
Makuyu, Kenya	S105 Brown	*Go. zambesina*	99.54	SAPBA773-07 (Kenya)
Makuyu, Kenya	S107 Brown	*Go. zambesina*	99.24	SAPBA773-07 (Kenya)
Botswana	IBB-1	*Go. belina*	99.84	SATWA003-06 (Zambia)
Botswana	IBB-2	*Go. belina*	99.85	SATWA003-06 (Zambia)
Kenya	GMB-2	*Gy. nigra*	100	GBMNC60687-20 (Botswana)
Botswana	GM-1	*Gy. westwoodi*	99.69	STBOA580-07 (Kenya)
Botswana	GM-2	*Gy. westwoodi*	99.69	STBOA580-07 (Kenya)

**Table 3:** Genetic p-distance comparisons for saturniid species.

Insect Species (Sample Size)	Genetic p-Distance Range within Sample Species	BOLD Sequence Used for Comparison (BOLD BIN Cluster Number)	Genetic p-Distance Range between Sample Species and BOLD Sequence
*Gonimbrasia zambesina* (15)	0–0.61	SAPBA773-07 (BOLD:AAD1339)	0–0.91
*Gonimbrasia krucki* (2)	0.0	SAPBA635-07 (BOLD:AAD8374)	0.0
*Gonimbrasia belina*-Kenya (1)	0.0	GBMNC60703-20 (BOLD:AAB6786)	0.0
*Gonimbrasia cocaulti* (7)	0–1.52	SPBIS152-09 (BOLD:AEH8028)	1.42–2.88
*Gonimbrasia belina*-Botswana (2)	0.0	SATWA003-06 (BOLD:AAB6786)	0.15–0.16
*Cirina forda* (4)	0.3–2.13	SATWA281-07 (BOLD:AAB6982)	0.46–2.13
*Bunaea alcinoe*-Kenya (8)	0–1.52	LSAFR2238-12 (BOLD:AAA6757)	0.61–1.98
*Bunaea alcinoe*-Nigeria (1)	0.0	SATWA891-07 (BOLD:AAA6756)	0.0
*Gynanisa nigra* (1)	0.0	STBOC836-08 (BOLD:AED6623)	0.61
*Gynanisa westwoodi* (2)	0.0	STBOA580-07 (BOLD:ABY4629)	0.3

**Table 4:** Seasonality and distribution of edible saturniids in Kenya.

Saturniid	Place Found	April–June	October–December
*Gonimbrasia zambesina*	Kilifi, Embu, Machakos, Kwale, Murang’a	Present	Present
*Cirina forda*	Kilifi, Nakuru, Embu, HomaBay, Kajiado	Present	Present
*Gonimbrasia cocaulti*	Taita, Makueni, Machakos, Kitui, Isiolo	Present	Absent
*Bunaea alcinoe*	Machakos, Makueni, Homabay, Meru, Kitui, Embu, Laikipia	Present	Present
*Gonimbrasia krucki*	Nairobi	Present	Present
*Gonimbrasia belina*	Kwale	Present	Present

**Table 5:** Area under curve values for training and test data.

Species	Current		RCP8.5	
	Training	Test	Training	Test
*Bunaea alcinoe*	0.855	0.915	0.877	0.928
*Cirina forda*	0.850	0.867	0.876	0.860

**Table 6:** Host plants consumed by edible saturniids in Kenya.

Saturniid	Host Plant
*Gonimbrasia zambesina*	*Mangifera indica, Anacardium occidentale*
*Cirina forda*	*Euclea divinorum, Acacia mearnsii, Manilkara sulcata*
*Gonimbrasia cocaulti*	*Vachellia tortilis, Vachellia nilotica*
*Bunaea alcinoe*	*Balanites aegyptiaca*, *Balanites glabra*
*Gonimbrasia krucki*	*Schinus terebinthifolia, Schinus molle*
*Gonimbrasia belina*	*Anacardium occidentale*

**Table 7:** Identities of edible saturniid host plants based on similarities with sequences from GenBank.

Collection Site	Sample Code	Species	% Similarity to GenBank Sequences	ID of Similar Sequences
Gilgil, Nakuru	HP7	*Euclea divinorum*	100	DQ924074.1
Mbita, Homabay	HP17	*Euclea divinorum*	97.06	DQ924074.1
Mbita, Homabay	HP18	*Euclea divinorum*	98.11	DQ924074.1
Gilgil, Nakuru	HP37	*Euclea divinorum*	99.64	DQ924074.1
Embu	HP38	*Euclea divinorum*	99.76	DQ924074.1
Gilgil, Nakuru	HP39	*Euclea divinorum*	100	DQ924074.1
Embu	HP40	*Euclea divinorum*	99.76	DQ924074.1
Matuu, Machakos	HP21	*Vachellia tortilis*	99.54	AF274140.1
Taita	HP22	*Vachellia tortilis*	99.77	AF274140.1
Makueni	HP23	*Vachellia tortilis*	99.77	AF274140.1
Mwingi	HP24	*Vachellia tortilis*	99.88	AF274140.1
Ngong, Kajiado	HP25	*Acacia mearnsii*	99.88	HM020723.1
Ngong, Kajiado	HP26	*Acacia mearnsii*	99.76	HM020723.1
Ngong, Kajiado	HP27	*Acacia mearnsii*	100	HM020723.1
Ngong, Kajiado	HP28	*Acacia mearnsii*	100	HM020723.1
Kilifi, Malindi	HP16	*Manilkara* sp.	99.40	DQ924092.1
Kilifi, Malindi	HP33	*Manilkara* sp.	99.40	DQ924092.1
Kilifi, Malindi	HP36	*Manilkara* sp.	99.40	DQ924092.1
Muhaka, Kwale	HP11	*Anacardium occidentale*	100	KY635877.1
Malindi, Kilifi	HP10	*Mangifera indica*	100	KX871231.1
Mbita, Homabay	HP3	*Balanites* sp.	99.35	JX517722.1
Mbita, Homabay	HP32	*Balanites* sp.	99.22	JX517722.1
Matuu, Machakos	HP45	*Balanites* sp.	99.48	JX517722.1
Matuu, Machakos	HP46	*Balanites* sp.	99.48	JX517722.1
Nanyuki	HP47	*Balanites* sp.	99.48	JX517722.1
Embu	HP48	*Balanites* sp.	99.48	JX517722.1
Embu	HP45	*Balanites* sp.	99.48	JX517722.1
Matuu, Machakos	HP2	*Vachelia nilotica*	99.30	KY10024.1
Buruburu, Nairobi	HP49	*Schinus terebinthifolia*	100	KP149521.1
Buruburu, Nairobi	HP51	*Schinus terebinthifolia*	100	KP149521.1
Kasarani, Nairobi	HP63	*Schinus terebinthifolia*	100	KP149521.1
Kasarani, Nairobi	HP62	*Schinus terebinthifolia*	100	KP149521.1
Kasarani, Nairobi	HP60	*Schinus terebinthifolia*	99.53	KP149521.1
Kasarani, Nairobi	HP61	*Schinus terebinthifolia*	99.53	KP149521.1
Kasarani, Nairobi	HP58	*Schinus terebinthifolia*	99.41	KP149521.1

## Data Availability

DNA sequences for species identification have been deposited in the GenBank (https://www.ncbi.nlm.nih.gov/WebSub/ (accessed on 10 March 2020)).

## Supplementary Materials

The following are available online at https://www.mdpi.com/article/10.3390/insects12070600/s1, Table S1. Location and agro-ecological zones of the study sites in Kenya; Table S2. Accession numbers of saturniid sequences submitted to the GenBank. Table S3. Pairwise genetic distances of *Gonimbrasia zambesina* samples. Table S4. Pairwise genetic distances of *Bunaea alcinoe* samples.
