# Supplementary material for: Diversity, Host Plants and Potential Distribution of Edible Saturniid Caterpillars in Kenya
Source: Insects. 2021 Jul 1;12(7):600. doi: 10.3390/insects12070600 (PMC8305150; doi:10.3390/insects12070600)
Supplement: Supplementary file 1 [file insects-12-00600-s001.zip › insects-1192598-supplementary.pdf]

Supplementary Materials

Table S1. Location and agro-ecological zones of the study sites in Kenya.

Study Site	GPS co-ordinates	Agro-ecological Zone
Gilgil, Nakuru	0.4923° S, 36.3173° E	Zone III
Mbeere, Embu	0.5388° S, 37.4596° E	Zone II
Nkubu, Meru	0.0647° S, 37.6679° E	Zone II
Nanyuki, Laikipia	0.0074° N, 37.0722° E	Zone IV
Kasarani, Nairobi	1.2254° S, 36.8976° E	Zone II
Kibwezi, Makueni	2.4105° S, 37.9678° E	Zone V
Kishushe, Taita	3.3973° S, 38.5559° E	Zone V
Muhaka, Kwale	4.2879° S, 39.5653° E	Zone IV
Malindi, Kilifi	3.2192° S, 40.1169° E	Zone V
Mbita, Homabay	0.4368° S, 34.2060° E	Zone III
Mwingi, Kitui	0.9374° S, 38.0605° E	Zone V
Ngong, Kajiado	1.3562° S, 36.6688° E	Zone III
Matuu, Machakos	1.1407° S, 37.5481° E	Zone IV
Isiolo, Isiolo	0.3556° N, 37.5833° E	Zone IV
Makuyu, Murang'a	0.9026° S, 37.1875° E	Zone II

Table S2. Accession numbers of saturniid sequences submitted to the GenBank.

	Sample	Accession Number
1	SUB7133105 S17_Gzambesina_Kilifi	MT176774
2	SUB7133105 S18_Gzambesina_Kilifi	MT176775
3	SUB7133105 S79_Gzambesina_Kambiti	MT176776
4	SUB7133105 S95_Gzambesina_Embu	MT176777
5	SUB7133105 S97_Brown_Gzambesina_Makuyu	MT176778
6	SUB7133105 S98_Brown_Gzambesina_Makuyu	MT176779
7	SUB7133105 S99_Green_Gzambesina_Makuyu	MT176780
8	SUB7133105 S100_Green_Gzambesina_Makuyu	MT176781
9	SUB7133105 S101_Green_Gzambesina_Makuyu	MT176782
10	SUB7133105 S102_Green_Gzambesina_Makuyu	MT176783
11	SUB7133105 S104_Brown_Gzambesina_Makuyu	MT176784
12	SUB7133105 S105_Brown_Gzambesina_Makuyu	MT176785
13	SUB7133105 S107_Brown_Gzambesina_Makuyu	MT176786
14	SUB7133102 S48_Gufipana_Muhaka	MT176766
15	SUB7108149 S22_Gcocaulti_Matuu	MT159807
16	SUB7108149 S28_Gcocaulti_Matuu	MT159808
17	SUB7108149 S30_Gcocaulti_Mwingi	MT159809
18	SUB7108149 S32_Gcocaulti_Taita	MT159810
19	SUB7108149 S33_Gcocaulti_Taita	MT159811
20	SUB7108149 S34_Gcocaulti_Taita	MT159812
21	SUB7108145 IBB1_Gbelina	MT157403
22	SUB7108145 IBB2_Gbelina	MT157404
23	SUB7133102 S48_Gufipana_Muhaka	MT176766
24	SUB7074604 Nigeria1_Balcinoe	MT179695
25	SUB7074604 S84_Balcinoe_Matuu	MT179696
26	SUB7074604 S85_Balcinoe_Matuu	MT179697
27	SUB7074604 S87_Balcinoe_Nanyuki	MT179698
28	SUB7074604 S88_Balcinoe_Nanyuki	MT179699
29	SUB7074604 S90_Balcinoe_Mbita	MT179700
30	SUB7074604 S91_Balcinoe_Mbita	MT179701
31	SUB7074604 S92_Balcinoe_Embu	MT179702
32	SUB7074604 S93_Balcinoe_Embu	MT179703

33	SUB7074617 2CF_Cforda_Ngong	MT179704
34	SUB7074617 5CF_Cforda_Ngong	MT179705
35	SUB7074617 S6_Cforda_Mbita	MT179706
36	SUB7074617 S7_Cforda_Mbita	MT179707
37	SUB7074617 S54_Cforda_Gilgil	MT179708
38	SUB7074617 S55_Cforda_Kilifi	MT179709
39	SUB7138372 GM1_Gwestwoodi	MT182818
40	SUB7138372 GM1_Gwestwoodi	MT182818
41	SUB7138303 GMB2_Gnigra	MT179594
42	SUB7133097 S2_Gkrucki_Nairobi	MT178412
43	SUB7133097 S3_Gkrucki_Nairobi	MT178413

Table S3. Pairwise genetic distances of *Gonimbrasia zambesina* samples.

Samples	KAG94	EMG95	MAB97	MAB98	MAG99	MAG100	MAG101	MAG102	MAB104	MAB105	MAB106	MAB107	KFG17	KFG18	KAG79	SAPBA773-07	SAPBA772-07
KAG94	0.00%																
EMG95	0.00%	0.00%															
MAB97	0.15%	0.15%	0.00%														
MAB98	0.15%	0.15%	0.00%	0.00%													
MAG99	0.15%	0.15%	0.00%	0.00%	0.00%												
MAG100	0.15%	0.15%	0.00%	0.00%	0.00%	0.00%											
MAG101	0.30%	0.30%	0.15%	0.15%	0.15%	0.15%	0.00%										
MAG102	0.30%	0.30%	0.15%	0.15%	0.15%	0.15%	0.31%	0.00%									
MAB104	0.30%	0.30%	0.15%	0.15%	0.15%	0.15%	0.31%	0.31%	0.00%								
MAB105	0.15%	0.15%	0.00%	0.00%	0.00%	0.00%	0.15%	0.15%	0.15%	0.00%							
MAB106	0.15%	0.15%	0.00%	0.00%	0.00%	0.00%	0.15%	0.15%	0.15%	0.00%	0.00%						
MAB107	0.30%	0.30%	0.15%	0.15%	0.15%	0.15%	0.31%	0.31%	0.31%	0.15%	0.15%	0.00%					
KFG17	0.61%	0.61%	0.46%	0.46%	0.46%	0.46%	0.61%	0.61%	0.61%	0.46%	0.46%	0.61%	0.00%				
KFG18	0.61%	0.61%	0.46%	0.46%	0.46%	0.46%	0.61%	0.61%	0.61%	0.46%	0.46%	0.61%	0.00%	0.00%			
KAG79	0.15%	0.15%	0.00%	0.00%	0.00%	0.00%	0.15%	0.15%	0.15%	0.00%	0.00%	0.15%	0.46%	0.46%	0.00%		
SAPBA773-07	0.61%	0.61%	0.46%	0.46%	0.46%	0.46%	0.61%	0.61%	0.61%	0.46%	0.46%	0.61%	0.00%	0.00%	0.46%	0.00%	
SAPBA772-07	1.22%	1.22%	1.07%	1.07%	1.07%	1.07%	1.23%	1.23%	1.23%	1.07%	1.07%	1.23%	1.22%	1.22%	1.07%	1.22%	0.00%

Table S4. Pairwise genetic distances of *Bunaea alcinoe* samples.

Samples	NGR1	SATWA891-07.COI-5P	MAB84	MAB85	MAB86	NAB87	NAB88	MBB90	MBB91	EMB92	EMB93	LSAFR2238-12
NGR1	0.00%											
SATWA891-07.COI-5P	0.00%	0.00%										
MAB84	3.60%	3.60%	0.00%									
MAB85	3.44%	3.44%	0.15%	0.00%								
MAB86	3.44%	3.44%	0.15%	0.00%	0.00%							
NAB87	3.28%	3.28%	0.30%	0.15%	0.15%	0.00%						
NAB88	3.28%	3.28%	0.30%	0.15%	0.15%	0.00%	0.00%					
MBB90	3.44%	3.44%	0.15%	0.00%	0.00%	0.15%	0.15%	0.00%				
MBB91	3.45%	3.45%	0.46%	0.31%	0.31%	0.15%	0.15%	0.31%	0.00%			
EMB92	3.28%	3.28%	0.31%	0.15%	0.15%	0.00%	0.00%	0.15%	0.15%	0.00%		
EMB93	3.76%	3.76%	0.77%	0.61%	0.61%	0.46%	0.46%	0.61%	0.61%	0.46%	0.00%	
LSAFR2238-12	3.76%	3.76%	0.76%	0.61%	0.61%	0.76%	0.76%	0.61%	0.92%	0.76%	1.23%	0.00%
